# Prevalence and distribution of M-proteins in the oncologic population affected by solid tumor

**DOI:** 10.1038/s41408-024-01095-7

**Published:** 2024-07-22

**Authors:** Renato de Falco, Giulia Togo, Anita Minopoli, Marco Cuomo, Domenica Rea, Serena Meola, Ernesta Cavalcanti

**Affiliations:** 1 Laboratory Medicine Unit, Istituto Nazionale Tumori - IRCCS - Fondazione “G. Pascale”, Naples, Italy; 2 Maxillofacial Surgery Unit, Department of Neurosciences, Reproductive and Odontostomatological Sciences, University Federico II, Naples, Italy

**Keywords:** Cancer, Epidemiology, Haematological cancer

Dear Editor,

Monoclonal Gammopathies (MG) are characterized by the proliferation of monoclonal plasma cells in the bone marrow with abnormal secretion of monoclonal immunoglobulins (M-proteins). MG include malignant disorders, such as Multiple Myeloma and Waldenström Macroglobulinemia and premalignant conditions, such as Smoldering Multiple Myeloma and Monoclonal Gammopathies of Undetermined Significance [1].

Several studies have already assessed the prevalence and distribution of MG in a healthy population and relatives of hematological patients [2, 3]. A slightly higher risk of solid tumors in patients with MGUS is reported in literature [4–6], meanwhile fewer data exist on the prevalence of MG in patients with solid tumors [7, 8]. The aim of this study is to verify and describe the prevalence and distribution of M-protein in an oncologic population affected by solid tumor.

All patients affected by solid tumor admitted to the Istituto Nazionale Tumori IRCCS Fondazione “G. Pascale” (Naples, Italy) between 2020 and 2022 (*n* = 14.626) received a serum capillary electrophoresis performed by CAPILLARYS 2 (Sebia, Cedex, France). Serum immunofixation electrophoresis was performed to confirm and identify the type of M-protein using the agarose HYDRAGEL IF 4 kit on HYDRASIS 2 (Sebia). The measurement of urinary M-protein or serum light-chains were not performed, because these tests are usually only required for hematological patients, who were excluded in our study. The study was performed in accordance with the revised version of the Declaration of Helsinki.

The mean ± Standard Deviation was used to describe parametric continuous variable, the median and interquartile range (IQR) were assessed for non-parametric continuous variable while counts and percentages were used for categorical variables. Differences between groups normally distributed with homogeneity of variance were analyzed with Student’s t test or Mann-Whitney test as appropriate. Exact 95% Confidence Intervals (CI) for prevalence were computed using the binomial distribution. Difference between 2 × 2 categorical variables were analyzed with Fisher’s exact test. Logistic regression was used to consider the weight of each risk factor. A forward stepwise selection based on Wald statistics was adopted with 0.05 and 0.10 enter and remove significance level respectively. All tests conducted were two tailed, and *P* values < 0.05 were considered statistically significant. Statistical analyses were performed with SPSS 28.0 software (SPSS, Chicago, IL, USA).

The characteristics of the evaluated population are described in Supplementary Table 1. The mean age was 59.7 ± 15.6 years and age difference between man and female was evaluated (62.1 ± 15.0 vs 57.6 ± 15.8, *P* < 0.001). The population was also stratified and divided in age groups (<40 years, 40–49 years, 50–59 years, 60–69 years, 70–79 years and ≥80 years) and a significant difference in age groups was found between men and women (*P* < 0.001).

M-proteins were detected in 336 patients, with an overall prevalence of 2.3%, which aligns with the previously reported prevalence in a healthy population [2], with a median concentration of 0.36 (IQR 0.25–0.54) mg/dL. In both sexes, prevalence increased with age (*P* < 0.001) and a higher prevalence of M-proteins was detected in men compared to women (2.9% vs 1.8%, *P* < 0.001). Therefore, the higher MG prevalence in older age and male gender described in literature was confirmed in our population (Fig. 1, Supplementary Table 2).

**Fig. 1:**
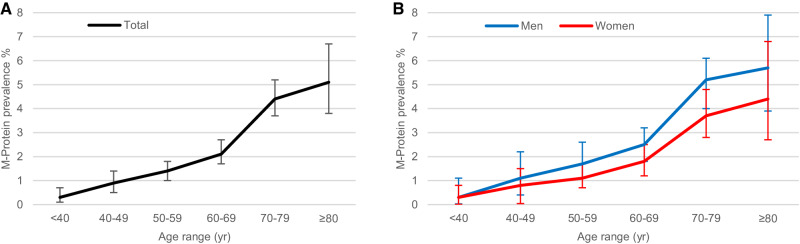
Prevalence of M-protein according to age and sex groups. **A** Prevalence of M-protein according to age groups in the oncologic population. **B** Prevalence of M-protein according to age and sex groups in the oncologic population. The I bars represent 95% confidence intervals.

M-protein types are described in Table 1. We performed serum immunofixation electrophoresis on 336 patients and we found IgG isotype in 218 patients (64.9%), IgM in 58 patients (17.2%), IgA in 45 patients (13.4%), biclonal M-protein in 15 patients (4.5%). We found kappa light-chain in 204 patients (60.7%) and lambda light-chain in 123 patients (36.9%), while 8 patients (2.4%) with biclonal M protein showed both kappa and lambda light chain. These distributions were similar to those reported in a healthy population [3]. Moreover, according to literature [9], IgM isotype was more expressed in men than women (*P* = 0.018). We also found a higher frequency of kappa light-chain in men compared to women (66.2% vs 53.2%, *P* = 0.021).

**Table 1:** Comparison of M-protein type between men and women.

	Total	Men	Women	*P*
M-proteins (%)	336	195 (58.2)	141 (41.8)	<0.001
Isotype				
IgG (%)	218 (64.9)	121 (62.1)	97 (68.8)	ns
IgA (%)	45 (13.4)	23 (11.8)	22 (15.6)	ns
IgM (%)	58 (17.2)	42 (21.5)	16 (11.3)	0.018
Biclonal (%)	15 (4.5)	9 (4.6)	6 (4.3)	ns
Light Chain				
κ (%)	204 (60.7)	129 (66.2)	75 (53.2)	0.021
λ (%)	124 (36.9)	62 (31.8)	62 (44.0)	ns
κ + λ (%)	8 (2.4)	4 (2.0)	4 (2.8)	ns

Variables are reported as number (percentage). Differences between two groups were assessed using Fisher’s exact test.

We analyzed the prevalence of M-protein in different types of solid cancer (Supplementary Fig. 1, Supplementary Table 3) and a higher prevalence of M-protein was found in patients affected by lung cancer (3.7% vs 2.2%, *P* < 0.001). M-protein concentration in lung cancer patients showed a median of 0.32 (IQR 0.23–0.50) mg/dL, with no difference from patients affected by other solid tumors, who showed a median of 0.36 (IQR 0.25–0.56) mg/dL. M-protein types did not differ between patients affected by lung cancer and those affected by other solid tumors (Supplementary Table 4). Since the age of patients affected by lung cancer patients was different than the age of patients affected by solid tumor of other anatomical regions (64.05 ± 12.92 vs 59.32 ± 15.74, *P* < 0.001), logistic regression was carried out to assess the effect of age, gender and lung cancer on the likelihood of positive M-protein. We found that older age (OR 1.055, 95%CI 1.045–1.065, *P* < 0.001), male sex (OR 1.321, 95%CI 1.057–1.650, *P* = 0.014) and lung cancer (OR 1.460, 95%IC 1.054–2.023, *P* = 0.023) were independent risk factor for M-protein presence.

A slightly higher incidence of lung cancer in patients with MG has already been reported [4, 5], whereas the incidence of MG in lung cancer patients has been limitedly explored [7, 8]. The higher prevalence reported in our study could be due to the use of immunotherapy in lung cancer patients.

Moreover, we found 47 M-proteins in 2100 patients affected by Head and Neck tumor (2.24%), and only 2 IgA isotype, a notably lower frequency compared to IgA isotype prevalence in patients affected by solid tumor of other anatomical regions (4.3% vs 15.2%, *P* = 0.04). This finding may lead to further studies as IgA play a role in Head and Neck cancer [10, 11].

In conclusion, our results showed that the prevalence and distribution of M-proteins in patients affected by solid tumor are similar to those previously reported in the healthy population. Moreover, we highlighted how patients affected by lung cancer showed a 46% increased risk of developing MG and how a lower percentage of IgA isotype was identified in patients with Head and Neck cancer.

### Supplementary information

Supplemental Material

Supplementary Data Legend

## Data Availability

The datasets generated and analysed during the current study are available in the Zenodo repository (https://zenodo.org/records/10715118).
