# Supplementary material for: Prevalence and distribution of M-proteins in the oncologic population affected by solid tumor
Source: Blood Cancer J. 2024 Jul 22;14(1):121. doi: 10.1038/s41408-024-01095-7 (PMC11263620; doi:10.1038/s41408-024-01095-7)
Supplement: Supplementary file 1 — Supplemental Material [file 41408_2024_1095_MOESM1_ESM.pdf]

Supplementary Table 1

	Total	Men	Women	P
Number of Patients (%)	14 626	6 750 (46,2)	7 876 (53,8)	*< 0,001*
Age	59,7 ±15,6	62,1 ±15,0	57,6 ±15,8	*< 0,001*
< 40 years (%)	1 735 (11,9)	629 (9,3)	1 106 (14,0)	*< 0,001*
40 - 49 years (%)	1 879 (12,8)	654 (9,7)	1 225 (15,6)	*< 0,001*
50 - 59 years (%)	3 110 (21,3)	1 271 (18,8)	1 839 (23,3)	*< 0,001*
60 - 69 years (%)	3 701 (25,3)	1 916 (28,4)	1 785 (22,7)	*< 0,001*
70 - 79 years (%)	3 185 (21,8)	1 715 (25,4)	1 470 (18,7)	*< 0,001*
≥ 80 years (%)	1 016 (6,9)	565 (8,4)	451 (5,7)	*< 0,001*

Differences between two groups were assessed using Student T test or Fisher’s exact test as appropriate. The continuous parametric variable is reported as Mean ± Standard Deviation. Categorical variables are reported as number (percentage).

Supplementary Table 2

Age	Number of M-Protein	Prevalence (CI)	Prevalence in men (CI)	Prevalence in women (CI)
<40 years	5/1 735	0,3% (0,1% - 0,7%)	0,3% (0,03% - 1,1%)	0,3% (0,01% - 0,08%)
40-49 years	17/1 879	0,9% (0,5% - 1,4%)	1,1% (0,4% - 2,2%)	0,8% (0,04% - 1,5%)
50-59 years	42/3 110	1,4% (1,0% - 1,8%)	1,7% (1,1% - 2,6%)	1,1% (0,7% - 1,7%)
60-69 years	79/3 701	2,1% (1,7% - 2,7%)	2,5% (1,8% - 3,2%)	1,8% (1,2% - 2,5%)
70-79 years	140/3 185	4,4% (3,7% - 5,2%)	5,0% (4,0% - 6,1%)	3,7% (2,8% - 4,8%)
≥80 years	52/1 016	5,1% (3,8% - 6,7%)	5,7% (3,9% - 7,9%)	4,4% (2,7% - 6,8%)

Supplementary Figure 1

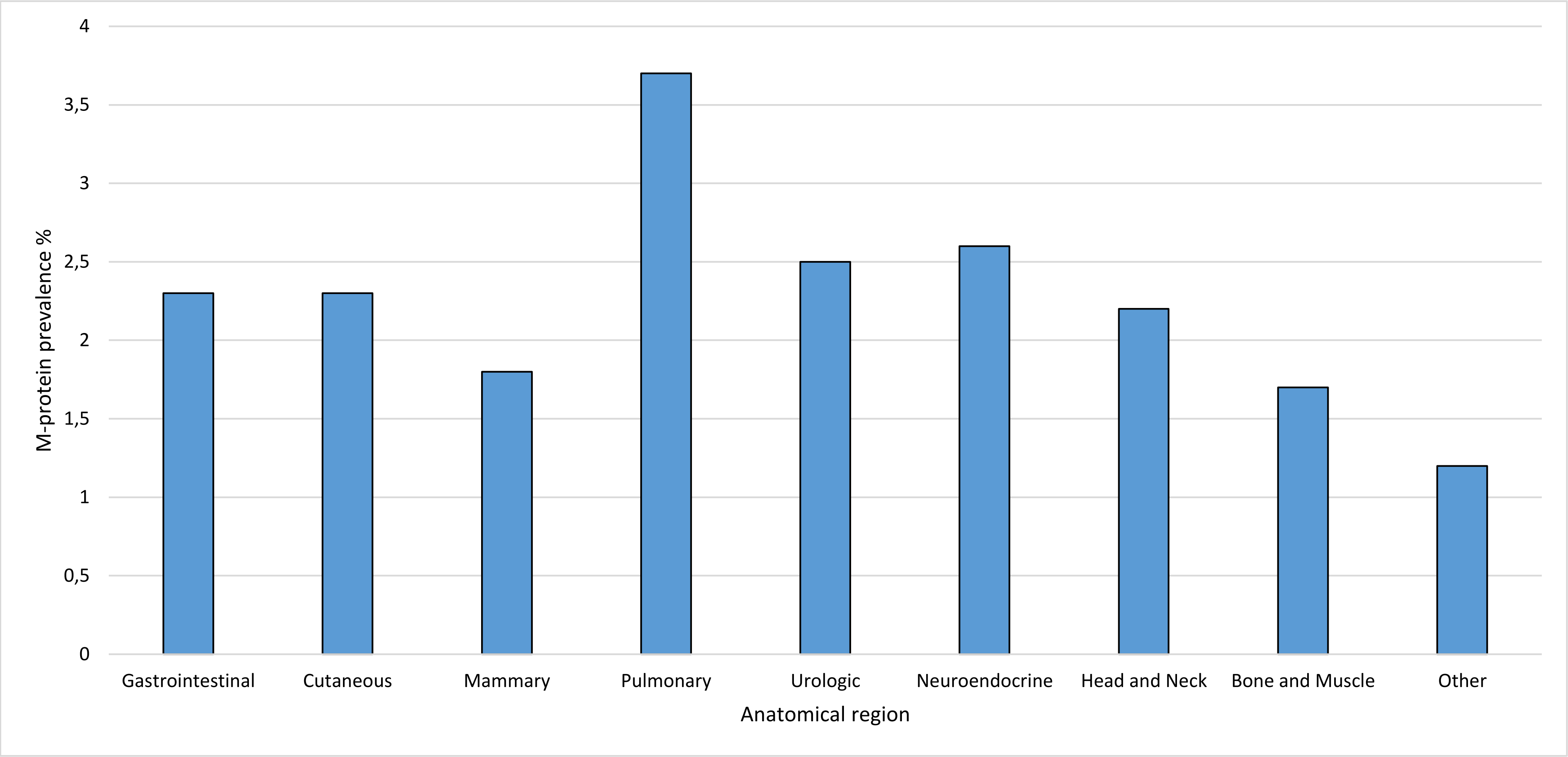

Supplementary Table 3

Anatomical Region	Number of M-Protein	Prevalence (CI)
Gastrointestinal	83/3647	2,3% (1,8% - 2,8%)
Cutaneous	24/1 054	2,3% (1,5% - 3,4%)
Mammary	47/2 629	1,8% (1,3% - 2,4%)
Pulmonary	44/1 176	3,7% (2,7% - 5,0%)
Urologic	76/3 058	2,5% (2,0% - 3,1%)
Neuroendocrine	5/196	2,6% (0,8% - 5,9%)
Head and Neck	46/2 100	2,2% (1,6% - 2,9%)
Bone and Muscle	7/421	1,7% (0,7% - 3,4%)
Other	4/345	1,2% (0,3% - 2,9%)

Supplementary Table 4

	Lung cancer	Other solid tumors	P
Prevalence (CI)	3,6 (2,7% - 4,8%)	2,2 (1,9% - 2,4%)	ns
Isotype			
IgG (%)	25 (56,8)	193 (66,1)	ns
IgA (%)	7 (15,9)	38 (13,4)	ns
IgM (%)	10 (22,7)	48 (16,4)	ns
Biclonal (%)	2 (4,5)	13 (4,5)	ns
Light Chain			
κ (%)	29 (65,9)	174 (58,6)	ns
λ (%)	14 (31,8)	110 (37,7)	ns
κ + λ (%)	1 (2,3)	8 (2,7)	ns
Differences between two groups were assessed using Fisher's exact test.			
