# Supplementary material for: Prevalence and distribution of M-proteins in the oncologic population affected by solid tumor
Source: Blood Cancer J. 2024 Jul 22;14(1):121. doi: 10.1038/s41408-024-01095-7 (PMC11263620; doi:10.1038/s41408-024-01095-7)
Supplement: Supplementary file 2 — Supplementary Data Legend [file 41408_2024_1095_MOESM2_ESM.pdf]

Supplementary Table 1: Characteristics of the population

Supplementary Table 2: Prevalence of M-protein according to age and sex groups

Supplementary Figure 1: Prevalence of M-protein according to type of cancer

Supplementary Table 3: Prevalence of M-protein according to type of cancer

Supplementary Table 4: Distribution of M-protein according to type of cancer
